# Prevalence of autism spectrum disorder and autistic symptoms in a school‐based cohort of children in Kolkata, India

**DOI:** 10.1002/aur.1812

**Published:** 2017-05-25

**Authors:** Alokananda Rudra, Matthew K. Belmonte, Parmeet Kaur Soni, Saoni Banerjee, Shaneel Mukerji, Bhismadev Chakrabarti

**Affiliations:** ^1^ Centre for Autism, School of Psychology and Clinical Language Sciences University of Reading, Reading UK; ^2^ The Com DEALL Trust Bangalore Karnataka India; ^3^ School of Social Sciences, Nottingham Trent University Nottingham UK; ^4^ Creating Connections Kolkata West Bengal India; ^5^ Graduate School of Creative Art Therapies, University of Haifa Haifa Israel; ^6^ Department of Psychology Ben Gurion University of the Negev Beer‐Sheva Israel

**Keywords:** low and middle income countries, autism, children, epidemiology, prevalence, assessment

## Abstract

Despite housing ∼18% of the world's population, India does not yet have an estimate of prevalence of autism. This study was carried out to estimate the prevalence of autism in a selected population of school‐children in India. *N* = 11,849 children (mean age = 5.9 [*SD* = 1.3], 39.5% females) were selected from various school types from three boroughs in Kolkata, India. Parents/caregivers and teachers filled in the social and communication disorders checklist (SCDC). Children meeting cutoff on parent‐reported SCDC were followed up with the social communication questionnaire (SCQ). SCQ‐positive children were administered the autism diagnostic observation schedule (ADOS). Teacher report on SCDC was available on all 11,849 children. Parent‐report SCDC scores were obtained for 5,947 children. Mean scores on teacher SCDC were significantly lower than parent SCDC. Out of 1,247 SCDC‐positive children, 882 answered the SCQ, of whom 124 met the cutoff score of 15. Six of these children met criteria for autism, autism spectrum disorder (ASD), or broader autism spectrum on the ADOS. The weighted estimate of supra‐threshold SCQ scores was 3.54% (CI: 2.88–4.3%). The weighted prevalence estimate of positive scores (for broader autism spectrum + ASD + autism) was 0.23% (0.07–0.46%). As ∼20% children in this state are known to be out of the school system, and ASD prevalence is likely to be higher in this group, this estimate is likely to represent the lower‐bound of the true prevalence. This study provides preliminary data on the prevalence of broader‐spectrum autism and supra‐threshold autistic traits in a population sample of school children in Eastern India. ***Autism Res** 2017, 10: 1597–1605*. ©2017 The Authors Autism Research published by Wiley Periodicals, Inc. on behalf of International Society for Autism Research

## Introduction

Prevalence studies on autism spectrum disorders (ASD) have been carried out in more than 15 countries since 1966, largely in the western hemisphere. Figures for prevalence of ASD are essential to determine the economic burden, to help establish more effective infrastructure and public policy, and to target research. Estimates vary from 4.1 per 10,000 individuals in 1966 (UK) to as high as 113 per 10,000 (USA) individuals in 2014 according to region and time [Elsabbagh et al., 2012]_._ Changing definitions of ASD can account for some of this observed variability. Specifically within Asia, estimates vary widely across time and country (China: 0.003–0.17%, Japan: 0.011–0.21%, South Korea: 1.89%) [Kim et al., 2011; Sun & Allison, 2010]. Heterogeneity of screening and diagnostic tools (SDTs) used in Asia has contributed to this variability; eight screening instruments have been used for the 26 prevalence studies in Asia. Five studies in Japan have used an 18‐month health checklist (HC‐18) [Honda, Shimizu, Imai, & Nitto, 2005; Honda, Shimizu, Misumi, Niimi, & Ohashi, 1996; Kawamura, Takahashi, & Ishii, 2008; Sugiyama & Abe, 1989; Tanoue, Oda, Asano, & Kawashima, 1988]. In China, five studies used the Chinese autism behavior scale [Zhang & Ji, 2005], two used the translated version of the autism behavior checklist [Volkmar et al., 1988], and others used a translated version of the checklist for autism in toddlers [Baron‐Cohen et al., 2000; Wong et al., 2004]. A local version of Bryson's Screening Scale was used in Indonesia while an Iranian study used the Childhood Symptom Inventory‐4 [Ghanizadeh, 2008; Sprafkin, Gadow, Salisbury, Schneider, & Loney, 2002]. A recent Korean study used a translated and validated version of the autism spectrum screening questionnaire followed by autism diagnostic observation schedule (ADOS) and autism diagnostic interview‐revised (ADI‐R) to confirm diagnostic status [Kim et al., 2011]. The diversity of screening instruments and study designs can potentially account for some of the variance in estimated prevalence [Elsabbagh et al., 2012; Sun & Allison, 2010]. Population‐based studies in Asia since 2000 establish a median observed prevalence of 13.9 per 10,000 individuals [Elsabbagh et al., 2012]. Unfortunately, India is the largest exception to the list of countries with an estimate of prevalence of autism and suprathrehold autistic traits in the general population [Elsabbagh et al., 2012; Malhotra & Vikas, 2005; Sun & Allison, 2010]. One reason for this lacuna has been the lack of availability of translated and validated SDTs for autism, which has been addressed in our earlier work [Rudra et al., 2014]. Two other recently available scales developed in India are the INCLEN diagnostic tool for autism spectrum disorder [Juneja et al., 2014] and the Indian scale for assessment of autism [Chakraborty, Thomas, Bhatia, Nimgaonkar, & Deshpande, 2015]. Estimates drawn from studies in the UK and USA suggest that India could have more than 2 million people with ASD [Krishnamurthy, 2008]. Very few studies have been carried out in India to estimate ASD prevalence [Malhotra & Vikas, 2005]. These small‐scale, hospital‐based studies have reported varying estimates of the prevalence of autism in psychiatric outpatient samples, varying widely from 2.9% to 62.5% [Bharath, Srinath, Seshadri, & Girimji, 1997].

The diversity of culture and socio‐economic status (SES) in India may bear on the manifestation of the autism phenotype. Socioeconomic factors have been shown to influence autism prevalence in other countries [Rai et al., 2012]. Particularly in the case of social communicative deficits toward the Asperger end of the autism spectrum, symptoms manifest only against the background of cultural norms and therefore the very existence of some symptoms may be partially a function of culture [Belmonte, 2011]. As pointed out by an early study, some Indian paediatricians are not concerned with the delay of language until 3 years of age [Daley & Sigman, 2002].

A prevalence estimate of autism in India would not only help to understand the impact of the condition but also would accelerate the development of appropriate government policies and help provide a framework for future research. With this aim, this study carried out a multi‐stage screening and validation procedure to measure the prevalence of autism and suprathreshold autistic traits in a school‐based cohort in India. This study's age range of 3–8 years captures a high enough age when most autism‐relevant behavior are clearly manifested, while avoiding major compensatory changes [Lord, Rutter, & Le Couteur, 1994].

## Methods

### Sample

**Figure 1:**
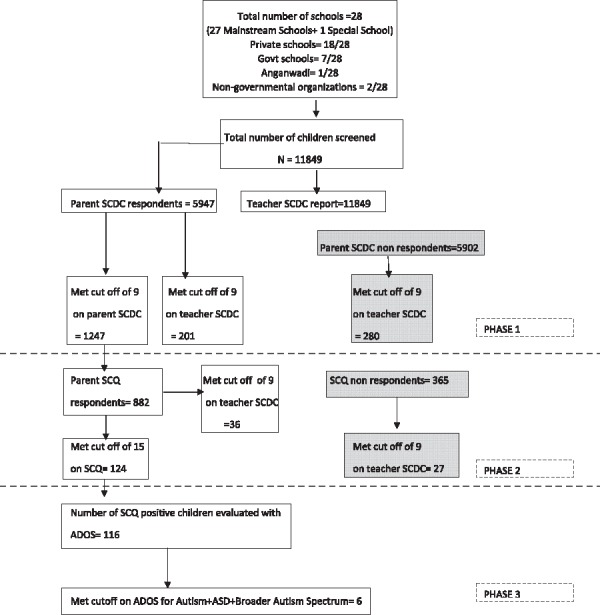
Flow chart showing the steps of the multi‐stage screening procedure for the study. Govt = government. Anganwadis are government childcare centers that provide supplementary nutrition, nonformal pre‐school education, nutrition and health education, health check‐ups, and referral services.

The study included children aged 3–8 years attending different types of schools in three boroughs or municipal wards (63, 64, and 65) in Kolkata (total population = 128,904). In most private schools in India, the age for entry is 2.5–3 years and above. However, in government schools, the age for entry can be as high as 6 years. The school types included were government (7 including central and state government schools), private (18), nongovernment organizations (2 mainstream schools were run by NGOs) and 1 group of anganwadi centers (Fig. 1). Anganwadis are government childcare centers that provide supplementary nutrition, nonformal pre‐school education, nutrition and health education, health check‐ups, and referral services. The centers are managed by trained workers. In ward 63, there were 11 anganwadi centers (no. 18–28), in 64, there were 14 centers (no. 140–153), and in 65, there were 13 centers (no. 141–149, 155 and 156 and 162–163). The set of all anganwadis was counted as one single school in the flowchart since each individual unit is significantly smaller than a school (Fig. 1). One special school for the hearing‐ and voice‐impaired fell within the selected catchment area and was included in the sample.

Of 29 schools approached, one school (partly government funded) refused participation on the basis of examinations being held during the time of this study. School fees were treated as an indicative proxy for SES, as detailed in Supporting Information Table S1.

The final sample size, though limited by the available resources, was chosen to be similar to that of the school‐based prevalence study of autism carried out in Cambridge in 2009 [Baron‐Cohen et al., 2009].

### Study Design

Ethical approval for this study was obtained from the Action for Autism Institutional Review Board (IRB). An information sheet with the details of the study and consent forms were distributed in all these schools. Written informed consent was obtained from parents willing to participate. Illiterate parents provided informed consent with their thumb print. Consent forms were returned to the school in sealed envelopes provided which were then collected by research assistants. The data were collected in three stages between 2010 and 2013, by a research team of postgraduate psychologists. The first and second stages took place from 2010 to 2012. The third stage, ADOS administration, was done in 2013. Data were collected from each school simultaneously.

#### Stage one

The first stage involved administration of the SCDC. SCDC is a short screening measure for autism comprising 12 questions pertaining to social and communicative behaviour. It was administered first because of its demonstrated efficiency as a quick screen for social reciprocity and communication in a general population (Skuse et al., 2005). A cutoff score of 9 was used, as suggested by a similar large‐scale study, to identify children with significant social communicative symptoms typically associated with ASD (Skuse et al., 2009). All teachers from the selected schools were provided with the social communication disorder checklist (SCDC) in hard copy, in English/Hindi/Bengali for each child eligible for enrolment. To facilitate quick completion of the questionnaire by the teacher, each class was represented by a spreadsheet with each of the SCDC items as a row, and each child as a column. Parents/caregivers filled in the SCDC in written form, or by telephone/in‐person interview, according to their preference. In low‐SES schools, teachers and anganwadi workers were asked to inform all the parents about the study. Parents were provided with information sheets (in Hindi and Bengali) and requested to come in for SCDC administration. Low‐SES parents/caregivers who were illiterate were administered the SCDC in person by research assistants. These parents were administered the SCDC for approximately 15 min, in groups of one to five.

#### Stage two

All parents/caregivers of children who scored above the threshold on the parent‐reported SCDC completed the social communication questionnaire (SCQ), a 40‐item parent‐reported screening tool for autism‐related traits, based on the ADI‐R [Berument, Rutter, Lord, Pickles, & Bailey, 1999]. The SCQ has been shown to aid clinicians in selecting preschool children who may show ASD traits, so as to diagnose them with the ADOS. The SCQ is highly sensitive in screening children of 2.5 years and older [Allen, Silove, Williams, & Hutchins, 2007; Corsello et al., 2007].

#### Stage three

Finally, all children who screened positive at the SCQ were administered the ADOS, by a research‐trained psychologist (S.M., trained for research administration of the ADOS at Great Ormond Street Hospital, London). ADOS administration took approximately 1 hr for each child. The ADOS is one of the most validated measures of autistic behavior, and involves a semi‐structured interview with quantitative coding [Rutter et al, 2002]. According to age range and verbal ability, children were administered either ADOS module two or three.

### Data analysis

Written responses were transcribed electronically, and statistical analysis was performed using SPSS version 19 (IBM Corp., Armonk, NY). For the SCDC, which comprises only 12 items, individuals with any missing items were excluded from further analysis. For the SCQ, omission of more than five items resulted in exclusion. SCQ scores with <5 missing items were extrapolated using the formula (total SCQ score + [mean item score x number of missing items]) similar to that used in earlier studies [Hoekstra, Bartels, Verweij, & Boomsma, 2007].

The unweighted estimate for suprathreshold SCQ scores was calculated by dividing the number of children who scored above the cutoff by the total number of children whose parents had completed the SCDC. The weighted prevalence estimate for suprathreshold SCQ scores was calculated after accounting for two factors: (a) the number of non‐respondents for SCQ among the individuals who scored above cutoff in the parent‐report SCDC, and (b) the base rate of suprathreshold teacher‐report SCDC score in the subset defined by point (a), using the formula below:

(1) P= ([SCQ+]∗[PSCDC+])/([SCQ)∗[PSCDC])

where, [SCQ+] = number of SCQ screen positive children; [PSCDC+] = number of parent‐report SCDC screen positive children; [SCQ] = number of SCQ respondents; [PSCDC] = number of parent‐report SCDC respondents.

This approach has the effect of dividing the unweighted estimate [SCQ+]/[PSCDC] by the SCQ response rate [SCQ]/[PSCDC+], thus increasing the estimate by the expected number of positives amongst the non‐responders.

The formula above assumes equal distribution of autistic traits among responders and non‐responders to parent‐report SCDC. To account for potentially different rates of suprathreshold autistic traits in non‐responders for parent‐report SCDC, a weighting factor *X* was calculated as follows:

(2) $$X=\left({\left[\text{TSCDC}+\right]}_{\text{PSCDCnr}}/{\left[\text{TSCDC}\right]}_{\text{PSCDCnr}}\right)/({\left[\text{TSCDC}+\right]}_{\text{PSCDCr}}/{\left[\text{TSCDC}\right]}_{\text{PSCDCr}})$$

where,

[TSCDC+]_PSCDCnr_ = number scoring above cutoff on teacher‐report SCDC among those who did not respond to parent‐report SCDC; [TSCDC]_PSCDCnr_ = number administered the teacher‐report SCDC among those who did not respond to the parent‐report SCDC; [TSCDC+]_PSCDCr_ = number scoring above cutoff on teacher‐report SCDC among those who responded to parent‐report SCDC; [TSCDC]_PSCDCr_ = number administered the teacher‐report SCDC among those who responded to the parent report SCDC

Using expressions (1) and (2), the final estimate of prevalence of suprathreshold SCQ scores was made using the following formula:

(3) $$P_{\text{weighted}}=\;\left(P*\left[\text{PSCDC}\right]+X*P*{\left[\text{TSCDC}\right]}_{\text{Pnonresp}}\right)/N_{\text{total}}$$

where *N*_total_ is the total number of children screened.

The estimates for the children meeting the cutoff for broader autism spectrum classification based on the ADOS were similarly calculated after accounting for the SCQ non‐respondents using the method described above (detailed formulae provided in Supporting Information). CIs for the key estimates of prevalence were calculated from 1,000 non‐parametric bootstrap samples, as done in a similar previous study [Baron‐Cohen et al., 2009].

## Results

### Sample Demographics

The sample from all 28 schools consisted of 11,849 children (mean age = 5.9 years, *SD* = 1.4). Teacher and parent report SCDC data were sought from all children in stage 1. Teacher report data were obtained on all children. Parent‐report SCDC was obtained from 5,947 children. Follow‐up of individuals who met cutoff on parent SCDC (*N* = 1,247) was done with the SCQ (*N* = 882). Of 124 children with suprathreshold scores on the SCQ, 116 took part in an ADOS administration.

Of those individuals who scored above cut‐off on the SCDC and thus were given the SCQ, 365 did not respond. Of 124 who were SCQ‐positive and invited for an ADOS administration, 8 did not respond. Children identified to be on the broader autism spectrum using the ADOS and clinical judgement was referred to the Mental Health foundation of India for further consultation. Unfortunately, due to limited resources, no follow‐up or referral was provided to non‐responders at any stage of the study. Sample demographics of teacher‐ and parent‐reported SCDC and SCQ are shown in Table 1. Supporting Information Table S2 shows the distribution of scores on each instrument for each SES. Supporting Information Table S3 describes the socio‐demographic profile of responders and non‐responders for each instrument.

**Table 1:** Sample Demographics and Mean Scores for All Instruments

		Parent SCDC	Teacher SCDC	SCQ
Overall	*N*	5,947	11,849	882
	Mean age (*SD*)	5.9 (1.4)	5.9 (1.3)	6.01(1.4)
	Mean score (*SD*)	5.04 (4.4)	1.04 (2.9)	9.6 (4.5)
Male	*N*	3,344	7,175	641
	Mean age (*SD*)	6.01 (1.2)	6 (1.2)	6.2 (1.3)
	Mean score (*SD*)	5.6 (4.6)	1.16 (3.1)	10.5 (4.4)
Female	*N*	2,603	4,674	241
	Mean age (*SD*)	5.8 (1.4)	5.8 (1.4)	5.9 (1.2)
	Mean score (*SD*)	4.29 (4.05)	0.85 (2.7)	7.4 (4.01)

### Missing Data

Teacher‐report SCDC data were available for non‐responders to parent‐report SCDC (*N* = 5,902) and parent‐report SCQ (*N* = 356). These data were used to adjust for potential differences in the distribution of suprathreshold autistic traits in the non‐responders, as described above and in the Supporting Information.

### Distributional Properties of SCDC and SCQ Scores

**Figure 2:**
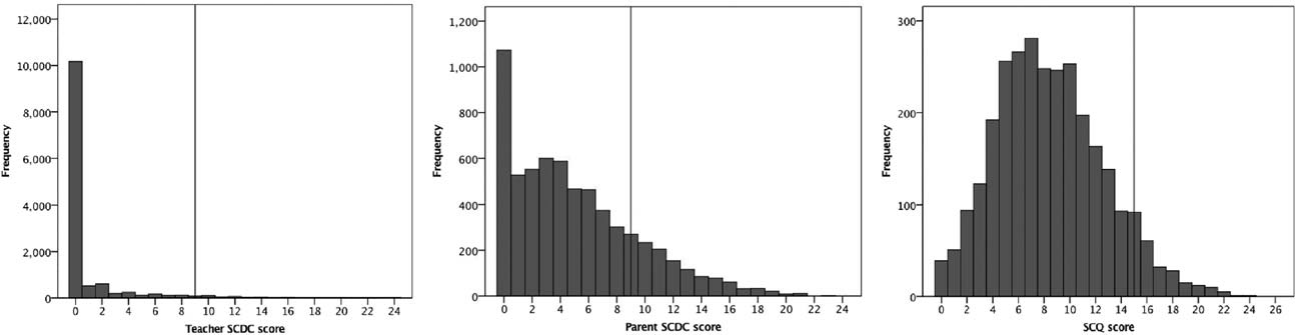
Histogram showing the distribution of the parent SCDC, teacher SCDC, and parent SCQ scores. Dotted line indicates cutoff scores on the respective instruments.

The Kolmogorov‐Smirnov test indicated significant deviation from normality (*P* < 0.001) for SCDC and SCQ data. Accordingly, nonparametric tests are reported in the following section. Figure 2 shows the distribution of the parent and teacher scores on SCDC and parent scores on SCQ. Cronbach's alpha for the SCDC in the current sample was 0.812. Cronbach's alpha for the SCQ in the current sample was 0.631.

### Responder and Gender Differences

Parents reported significantly greater SCDC scores than did teachers (Wilcoxon signed‐rank *Z* = 49.9, *r* = 0.7, *P* < 0.001). Teacher‐reported SCDC scores were greater for SCQ respondents in comparison to SCQ non‐respondents (*Z* = 4.8, *r* = 0.14, *P* < 0.001). Males were associated with significantly higher scores than females on all instruments (Table 1).

### Preliminary Prevalence Estimate

A preliminary prevalence estimate was calculated from the follow‐up of individuals with SCDC data from parents (Fig. 1). Out of 5,947 children whose parents completed the SCDC, 1,247 met the cutoff score of 9. Of these, 882 participated in the SCQ stage. One hundred and twenty‐four children met the SCQ cutoff of 15. Of these 124 SCQ‐positive children, 116 agreed to an ADOS administration. Of these 116 children, a total of 6 met the ADOS cutoff in different modules, and were clinically judged to demonstrate broader‐spectrum autism. One child met the ADOS cutoff for autism in Module 2 (total of social and communication scores ≥9, for children above 5 years). Two children met the cutoff for ASD in Module 3 (total of social and communication scores ≥7 and <10). One child met the broader autism spectrum cutoff in Module 2 (age above 5 years, total of social and communication scores ≥6 and <8) and two children met the cutoff in Module 3 (total of social and communication scores ≥5 and <7), using the criteria as defined in a recent large‐scale study in the general population [Colvert et al., 2015]. Similar results were observed across genders (Table 2).

**Table 2:** Number and Percentage of Individuals Scoring Above Cut‐Off on SCQ and ADOS

	Overall	Male	Female
Parent SCDC respondents	5,947	3,344	2,603
SCDC screen positive	1,247	844	403
SCQ respondents	882	641	241
SCQ screen positive	124	115	9
ADOS participants	116	109	7
ADOS (broader spectrum cutoff) positive	6	6	0
Weighted estimate of suprathreshold SCQ scores (%)	3.54	5.06	0.89
Weighted estimate of children meeting broader spectrum cutoff on ADOS (%)	0.23	0.33	0

The unweighted overall prevalence estimate for supra‐threshold SCQ scores was 2.09% (bootstrapped CI: 1.7–2.5%) and the weighted estimate was 3.54% (bootstrapped CI: 2.88–4.3%). The estimate of the children meeting criteria for broader autism spectrum (also including autism, and ASD) according to the ADOS and clinical judgement was calculated similarly, incorporating individuals who met cutoff scores on the SCQ and were administered the ADOS. The unweighted prevalence estimate for broader autism spectrum was 0.1% (bootstrapped CI: 0.03–0.18%), while the weighted estimate was 0.23% (bootstrapped CI: 0.07–0.46%). This figure was driven in equal measures by ASD per se and by the broader autism spectrum.

## Discussion

This study provides a preliminary estimate of the prevalence of broader autism spectrum and supra‐threshold autistic symptoms in a large school‐based sample from one city in eastern India. This study applies widely used, gold‐standard tools for autism screening and diagnosis, translated and validated in regional languages [Rudra et al., 2014]. As such, this study provides the first estimate of autism‐related symptoms in a large school‐going cohort in India. The choice to implement a school‐based design rather than a hospital register‐based design was necessitated by the lack of availability of central disability registers/hospital records with patient diagnosis details (as available in many Western countries). The school‐based design allows an advantage in being able potentially to flag higher‐functioning children, who often are not identified until a later age. However—as elaborated later—an inherent disadvantage of such designs is the lack of data from children who are outside the school system.

A number of studies have used similar designs involving multi‐stage screening of children [Baird et al., 2006; Baron‐Cohen et al., 2009; Kim et al., 2011]. The sample size of 11,849 children is comparable to that of the prevalence study carried out in mainstream schools in the UK (*N* = 11,635, 9–10 year‐olds) [Baron‐Cohen et al., 2009]. The 46.7% response rate obtained in this study exceeds that obtained in the UK study (30%) but is less than that obtained from the regular school population sample in the South Korean study (63%) [Kim et al., 2011].

This prevalence estimate of 0.23% for broader autism spectrum (based on ADOS) in school‐going children is likely to represent a lower‐bound of the true prevalence of ASD in this age group. This inference is driven by school enrolment data available from the Government of India (http://schoolreportcards.in/). West Bengal (the state of which Kolkata is the capital) had a net enrolment ratio of 79.4% for 6–11 year olds in the appropriate primary and upper primary schools in 2012–2013. It is possible that there is a higher prevalence of autism (as well as other mental and physical health conditions) in the ∼20% of children who are outside the school system. Of all the children enrolled in schools, only 0.008% reported a clinical diagnosis of Autism. In comparison, Kerala (another state of India of a similar size, but with a higher net enrolment ratio [83%] and higher literacy rate [93.9%]) had a significantly higher percentage of children with reported Autism enrolled in schools (0.025%). If the true prevalence of ASD is assumed to be comparable across the two states, these data indicate that about one third of the children with ASD are entering the school system in West Bengal, compared to that in Kerala.

This low estimate of prevalence in our study is however comparable to several other population‐based prevalence estimates in other Asian countries. In a similar study carried out in TAIF‐KSA (Saudi Arabia) in a school‐going population (7–12 years of age), overall prevalence was very low (0.035%) [Al‐Zahrani, 2013]. An Indonesian study reported a prevalence of 0.117% for ASD [Wignyosumarto, Mukhlas, & Shirataki, 1992]. A subsequent study in China reported a similar figure of 0.11% (11 per 10,000 children) for autistic disorder [Zhang & Ji, 2005]. Wong and Hui, using government population statistics for ASD, noted an estimated 5‐year incidence of 7.9 per 10,000 for children under 5 years in Hong Kong in the period 2001–2005 [Wong & Hui, 2007]. In Iran, a prevalence of 0.063% was reported for typical autism [Samadi, Mahmoodizadeh, & McConkey, 2012]. In Japan, cumulative incidence and prevalence of high functioning childhood autism were 0.162% and 0.211%, respectively [Honda et al., 1996]. Other than the study in Saudi Arabia, these were population‐based studies on school‐age children.

The present results are in line with rates observed in some Asian countries but lower than those reported in other studies [Baird et al., 2006; Kim et al., 2011; Wong & Hui, 2007]. Such reports of low incidence of ASD in Asian countries (except South Korea, Kim et al., 2011) are of particular significance for exploring cultural factors that may influence identification of ASD.

Many moderately to severely affected children, in particular, might escape notice in such school‐based population studies, because they are not enrolled in school. For children with a diagnosis who are currently enrolled in schools, it is possible that their parents choose not to respond to such surveys because of the stigma associated with psychiatric diagnoses. However, this possibility is low in the current sample, since we observed that the mean teacher‐reported SCDC scores for SCQ respondents exceeded those for SCQ non‐respondents. Though studies carried out in preschool suggest a lower cutoff for SCQ, a cutoff of 15 was used to identify children with suprathreshold autism‐related traits as suggested in the original paper [Berument et al., 1999] and previously validated with our own translation of the SCQ [Rudra et al, 2014]. In this study, the distribution of the SCQ scores (Fig. 2), along with the parents' tendency to over‐endorse autistic traits, argues for adherence to this standard, validated screening cutoff.

Due to the absence of any prior available medical information on the children in this sample, we were unable to test for false negative rates. We tried to minimize this confound to an extent by basing calculations on both parent‐report and teacher‐report data. This strategy ensured that our estimate was not unduly influenced by parents who might not report autism‐relevant traits because of fear of stigmatization or diagnostic labelling.

The weighted estimate of 3.54% of children meeting supra‐threshold scores on the SCQ is closer to the estimate for ASD obtained in the South Korean study of 1.89% (CI = 1.43–2.36) as well as a study in Toyota, Japan by Kawamura et al. on PDD prevalence (1.81%, 95% CI = 1.6–2.06) [Kawamura et al., 2008; Kim et al., 2011]. In this study, a large difference in prevalence estimates is noticed when considering parent report data on their own, versus after including data from the ADOS administered by a research‐trained psychologist. The difference in prevalence estimates from parent SCQ data and ADOS administration highlights the over‐endorsement of ASD symptoms by parents. This observation is further supported by comparing the large mean SCDC scores from parents with the smaller ones from teachers. Murray et al. have pointed out that certain social skills are context‐dependent and their interpretation may vary between teachers and parents [Murray, Ruble, Willis, & Molloy, 2009]. Another possible source of this difference between parent and teacher responses might be the fact that parents have a much smaller set of children on whom to base their judgements. Over‐reporting of autistic traits by parents in India has been observed in a recent report [Venkatesan, 2015].

Males scored higher on both the SCDC and SCQ compared to females. None of the females met cutoff scores on the ADOS. This result reinforces the observation that males score higher than females on autistic trait measures [Auyeung, Baron‐Cohen, Wheelwright, & Allison, 2008; Valla et al., 2010]. The results also fit the trend for gender differences observed in previous studies on validation of autism screening tools and behavioral measures [Skuse et al., 2009].

Although a representative sample encompassing all levels of SES was approached for this study, limited resources for follow‐up and the low literacy rates in individuals from low‐SES accounted for lower participation from this group compared to that of middle and high SES. In future, this gap needs to be addressed through appropriate awareness drives and greater resource allocation for low‐SES data collection. In order to prevent stigmatization of children identified with broader autism spectrum in our study and to minimize the dropout rate, parents were informed that this was a developmental study of children, with no reference to autism. All personal information was kept confidential under a written agreement from each school that the results could only be divulged to the parents/caregivers of the individual child. Children who took part in the final (ADOS) stage of the study and were identified with any ASD were referred to the Mental Health Foundation of India for further consultation.

As a first study of its kind, this work is not without its limitations and hence caution is called for in interpreting its findings. One limitation is the potential under‐representation of the low‐SES children. Most low‐SES individuals may be daily wage earners and hence may have opted not to miss a day's earning. Second, illiterate parents at the low‐SES schools were administered the SCDC in groups of one to five. This group administration format may have resulted in biased answers. None of the parents of the three children who met cutoff on SCDC from low SES came forward to participate in the SCQ stage. This under‐representation of participants from the low SES may have led to a lower prevalence estimate. To understand the effect of this potential bias we reran the analysis without low SES individuals, which yielded a very similar weighted estimate for broader autism spectrum of 0.26%. As this is a small deviation, a substantial influence on our final estimate via such a mechanism seems unlikely.

The second limitation was the lack of access to disability registers from hospitals or to autism‐specific special schools in the catchment area. This study thus misses out on the chance to identify lower‐functioning children with autism, who might not be going to any school. Future studies should be conducted using a more extensive and representative sample (possibly using voter lists or door‐to‐door sampling).

## Conclusion

This study is one of the first to provide a preliminary estimate of the prevalence of broader autism spectrum and supra‐threshold autistic symptoms in an Indian school‐going population. The unweighted (0.1%) and weighted prevalence estimates for broader autism spectrum (0.23%) estimate should be considered with caution as they represent a lower‐bound of the true prevalence of ASD in the country. This result however can form the basis of future large‐scale epidemiological and related (genetic and environmental) research on autism in India. Such research in low‐resource settings is crucial in order to inform policies and to guide appropriate allocation of resources for individuals on the spectrum.

## Supporting information

Additional Supporting Information may be found in the online version of this article at the publisher's website.

**Table S1**. SES categorization according to school fees.

**Table S2**. Mean score obtained on all tools according to each SES category.
**Table S3**. Socio‐demographic profile of responders and non‐responders for each screening and diagnostic tool used in the study.
