# Supplementary material for: Prevalence of autism spectrum disorder and autistic symptoms in a school‐based cohort of children in Kolkata, India
Source: Autism Res. 2017 May 25;10(10):1597–605. doi: 10.1002/aur.1812 (PMC5655917; doi:10.1002/aur.1812)
Supplement: Supplementary file 1 — Table S1. SES categorization according to school fees. [file AUR-10-1597-s001.docx]

**SUPPLEMENTARY MATERIAL 1**

Proportion of children meeting suprathreshold SCQ scores is calculated using the following formula:

P= ([SCQ+]*[PSCDC+])/([SCQ)*[PSCDC]) …. …………………………………..(1)

where,

[SCQ+]= Number of SCQ screen positive

[PSCDC+] = Number of parent-report SCDC screen positive

[SCQ] = Number of SCQ respondents

[PSCDC] = Number of parent-report SCDC respondents

This approach has the effect of dividing the unweighted estimate [SCQ+]/[PSCDC] by the SCQ response rate [SCQ]/[PSCDC+], thus increasing the estimate by the expected number of positives amongst the non-responders.

The formula above assumes equal distribution of autistic traits among responders and non-responders to parent-report SCDC. To account for potentially different rates of suprathreshold autistic traits in non-responders for parent-report SCDC and SCQ, two weighting factors are calculated as follows.

X=([TSCDC+]_PSCDCnr_/[TSCDC]_PSCDCnr_)/ ([TSCDC+]_PSCDCr_/[TSCDC]_PSCDCr_ ) ……. (2)

where,

[TSCDC+]_PSCDCnr_=Number scoring above cutoff on teacher-report SCDC among those who did not respond to parent-report SCDC

[TSCDC]_PSCDCnr_= Number administered the teacher-report SCDC among those who did not respond to the parent-report SCDC

[TSCDC+]_PSCDCr_= Number scoring above cutoff on teacher-report SCDC among those who responded to parent-report SCDC

[TSCDC]_PSCDCr_= Number administered the teacher-report SCDC among those who responded to the parent report SCDC

Similarly, a weighting factor was calculated to account for differential rate of suprathreshold autistic traits in non responders for SCQ

Y= ([TSCDC+]_PSCQnr_/[TSCDC]_PSCQnr_)/([TSCDC+]_PSCQr_/[TSCDC]_PSCQr_)………….(3)

where,

[TSCDC+]_PSCQnr_=Number scoring above cutoff on teacher-report SCDC among those who did not respond to SCQ

[TSCDC]_PSCQnr_= Number administered the teacher-report SCDC among those who did not respond to SCQ

[TSCDC+]_PSCQr_= Number scoring above cutoff on teacher-report SCDC among those who responded to SCQ

[TSCDC]_PSCQr_= Number administered the teacher-report SCDC among those who responded to SCQ

Using expressions (1) and (2), the final estimate of prevalence of suprathreshold SCQ scores was made using the following formula:

P_weighted_= (P*[PSCDC]+X*P*[TSCDC]_Pnonresp_)/ N_total_ ……………………………….(4)

where N_total_ is the total number of children screened.

Similar to (1), the proportion of children meeting cutoff for broader ASD using the ADOS in SCQ respondents was calculated using the following formula:

Q= ([ADOS+]*[SCQ+])/([ADOS]*[SCQ]) ………………………………………………………………..….(5)

where

[ADOS+]= Number of children meeting cutoff on ADOS criteria for broader ASD

[ADOS] = Number of children administered the ADOS

Similar to (4), proportion of children meeting cutoff for broader ASD using the ADOS within PSCDC screen positive children was calculated as follows:

Z= (Q*[SCQ]+Y*Q*[TSCDC]_PSCQnr_)/[PSCDC] …………………………..(6)

Combining (4) and (6), the proportion of children meeting cutoff for broader ASD using the ADOS in the whole sample was calculated as follows:

Q_weighted_= (Q*[SCQ]+Y*Q*[TSCDC]_PSCQnr_+X*Z*[TSCDC]_PSCDCnr_)/ N_tot_ ................(7)

The above formulae were also applied to calculate prevalence estimates across genders.

Confidence intervals were calculated using 1000 nonparametric bootstrapped samples.

*Table 1: SES categorization according to school fees.*

| SES | NO.OF SCHOOLS | NO.OF CHILDREN | MEAN, ST.DEV ANNUAL FEES (NOMINAL VALUE) ($) | MEAN, ST.DEV ANNUAL FEES (PPP) ($) |
| --- | --- | --- | --- | --- |
| LOW | 9 | 1237 | No Fees | No Fees |
| MIDDLE | 14 | 7229 | 130.4 (27.9) | 434.8 (92.9) |
| HIGH | 5 | 3383 | 456.5 (35.7) | 1521.8 (119.01) |

**Conversion from rupees to dollars-* <http://www.rbi.org.in/home.aspx>

***Purchasing power parity in dollars-http://data.worldbank.org/indicator/PA.NUS.PPPC.RF*

*Table 2: Mean score obtained on all tools according to each SES category*

| SES |  | PARENT SCDC | TEACHER SCDC | SCQ |
| --- | --- | --- | --- | --- |
| LOW | N | 765 | 1237 | 0 |
|  | MEAN SCORE | 0.73 | 0.5 | NA |
|  | S.D | 1.5 | 2.1 | NA |
| MIDDLE | N | 3422 | 7229 | 743 |
|  | MEAN SCORE | 6.10 | 0.78 | 10.3 |
|  | S.D | 4.5 | 2.3 | 4.4 |
| HIGH | N | 1760 | 3383 | 170 |
|  | MEAN SCORE | 4.8 | 1.8 | 7.14 |
|  | S.D | 3.8 | 4.03 | 3.9 |

*Table 3- Socio-demographic profile of responders and non-responders for each screening and diagnostic tool used in the study.*

| PARENT TOOLS |  | LOW | MIDDLE | HIGH |
| --- | --- | --- | --- | --- |
| SCDC | Responders | 765 | 3422 | 1760 |
|  | Non responders | 472 | 3807 | 1623 |
| SCQ | Responders | 0 | 712 | 170 |
|  | Non responders | 3 | 229 | 133 |
| ADOS | Responders | 0 | 112 | 4 |
|  | Non responders | 0 | 7 | 1 |
