# Supplementary material for: Prevalence of autism spectrum disorder and autistic symptoms in a school‐based cohort of children in Kolkata, India
Source: Autism Res. 2017 May 25;10(10):1597–605. doi: 10.1002/aur.1812 (PMC5655917; doi:10.1002/aur.1812)
Supplement: Supplementary file 2 — Table S2. Mean score obtained on all tools according to each SES category. Table S3. Socio‐demographic profile of responders and non‐responders for each screening and diagnostic tool used in the study. [file AUR-10-1597-s002.docx]

SUPPLEMENTARY MATERIAL 2

MATLAB code used to calculate prevalence estimate and bootstrapped confidence intervals:

%%The un-weighted estimate for supra-threshold SCQ scores

% NPSCDC= 5947; %Numberof respondents on parent-report SCDC

% NSCQpos=124;  %Number of SCQ screen positive

% NPSCDCpos=1247; %Number of parent report SCDC screen positive

% NSCQ=882; %Number administered the teacher report SCDC who were SCQ respondents

% NSCQnonresp=365; % Number administered the teacher report SCDC who were SCQ non-respondents

% NPSCDC=5947; %Number of parent report SCDC respondents

% N= 11849; % Total Teacher SCDC respondents.

% TSCDCPresp= 5947; % Number administered the teacher report SCDC among those who responded to the parent report SCDC

% TSCDCposPnonresp=280;% Number scoring above cutoff on teacher report SCDC among those who did not respond to parent-report SCDC

% TSCDCPnonresp=5902;%Number administered the teacher report SCDC among those who did not respond to the parent report SCDC

% TSCDCposPresp= 201;% Number scoring above cutoff on teacher report SCDC among those who responded to parent-report SCDC

% TSCDCposPSCQresp=36; % Number scoring above cutoff on teacher report SCDC among those responded to parent-report SCQ

% NADOS= 116; % No. on whom ADOS was administered.

% NADOSpos= 6; % ADOS positive

% TSCDCposPSCQnonresp=27; % Number scoring above cutoff on teacher report SCDC among those did not respond to parent-report SCQ

[bootstat,bootsam] = bootstrp(1000,@mean,m); % We only use bootsam in this code.

N=zeros(1000,1);

tscdcpos=zeros(1000,1);

TSCDCposPresp=zeros(1000,1);

tscdcpospscdcpos=zeros(1000,1);

TSCDCposPSCQresp=zeros(1000,1);

NPSCDC=zeros(1000,1);

NPSCDCpos=zeros(1000,1);

NSCQ=zeros(1000,1);

NSCQpos=zeros(1000,1);

NADOS=zeros(1000,1);

adosposmod21=zeros(1000,1);

adosposmod22=zeros(1000,1);

adosposmod31=zeros(1000,1);

adosposmod32=zeros(1000,1);

for k=1:1000

for j=1:size(m,1)

    i=bootsam(j,k);

    if m(i,8)>=0 && m(i,9)== 0

        N(k)=N(k)+1;

    end

    if m(i,8)>=9 && m(i,9)== 0

        tscdcpos(k)=tscdcpos(k)+1;

    end

    if m(i,8)>=9 && m(i,9)== 0 && m(i,6)>=0 && m(i,7)==0

        TSCDCposPresp(k)=TSCDCposPresp(k)+1;

    end

     if m(i,8)>=9 && m(i,9)== 0 && m(i,6)>=9 && m(i,7)==0

        tscdcpospscdcpos(k)=tscdcpospscdcpos(k)+1;

     end

    if m(i,8)>=9 && m(i,9)== 0 && m(i,6)>=9 && m(i,7)==0 && m(i,10)>=0 && m(i,11)==0

        TSCDCposPSCQresp(k)=TSCDCposPSCQresp(k)+1;

    end

    if m(i,8)>=0 && m(i,9)== 0 && m(i,6)>=0 && m(i,7)==0

        NPSCDC(k)=NPSCDC(k)+1;

    end

    if m(i,8)>=0 && m(i,9)== 0 && m(i,6)>=9 && m(i,7)==0

        NPSCDCpos(k)=NPSCDCpos(k)+1;

    end

    if m(i,8)>=0 && m(i,9)== 0 && m(i,6)>=9 && m(i,7)==0 && m(i,10)>=0 && m(i,11)==0

        NSCQ(k)=NSCQ(k)+1;

    end

    if m(i,8)>=0 && m(i,9)== 0 && m(i,6)>=9 && m(i,7)==0 && m(i,10)>=15 && m(i,11)==0

        NSCQpos(k)=NSCQpos(k)+1;

    end

    if m(i,8)>=0 && m(i,9)== 0 && m(i,6)>=9 && m(i,7)==0 && m(i,10)>=15 && m(i,11)==0 && m(i,19)==1

        NADOS(k)=NADOS(k)+1;

    end

    if m(i,8)>=0 && m(i,9)== 0 && m(i,6)>=9 && m(i,7)==0 && m(i,10)>=15 && m(i,11)==0 && m(i,13)==2 && m(i,16)>=9 && m(i,12)>5

        adosposmod21(k)=adosposmod21(k)+1;

    end

     if m(i,8)>=0 && m(i,9)== 0 && m(i,6)>=9 && m(i,7)==0 && m(i,10)>=15 && m(i,11)==0 && m(i,13)==2 && m(i,16)>=6 && m(i,16)<8 && m(i,12)>5

        adosposmod22(k)=adosposmod22(k)+1;

     end

    if m(i,8)>=0 && m(i,9)== 0 && m(i,6)>=9 && m(i,7)==0 && m(i,10)>=15 && m(i,11)==0 && m(i,13)==3 && m(i,16)>=7 && m(i,16)<10

        adosposmod31(k)=adosposmod31(k)+1;

    end

    if m(i,8)>=0 && m(i,9)== 0 && m(i,6)>=9 && m(i,7)==0 && m(i,10)>=15 && m(i,11)==0 && m(i,13)==3 && m(i,16)>=5 && m(i,16)<7

        adosposmod32(k)=adosposmod32(k)+1;

    end

end

NADOSpos(k)=adosposmod21(k)+adosposmod22(k)+adosposmod31(k)+adosposmod32(k);

TSCDCposPSCQnonresp(k)=tscdcpospscdcpos(k)-TSCDCposPSCQresp(k);

TSCDCposPnonresp(k)=tscdcpos(k)-TSCDCposPresp(k);

NSCQnonresp(k)=NPSCDCpos(k)-NSCQ(k);

TSCDCPnonresp(k)=N(k)-NPSCDC(k);

TSCDCPresp(k)= NPSCDC(k);

UP_SCQ(k)= NSCQpos(k)/NPSCDC(k);%Unweighted_estimate_SCQ

UP_ADOS(k)= NADOSpos(k)/NPSCDC(k);%Unweighted Ados

%Proportion of children meeting suprathreshold SCQ scores

P(k)=NSCQpos(k)*(NPSCDCpos(k)/NSCQ(k))/NPSCDC(k);

X1(k)= (TSCDCposPnonresp(k)/TSCDCPnonresp(k))/(TSCDCposPresp(k)/TSCDCPresp(k));

X2(k)= (TSCDCposPSCQnonresp(k)/NSCQnonresp(k))/(TSCDCposPSCQresp(k)/NSCQ(k));

Final_estimate_supra_SCQ(k)= (P(k)*NPSCDC(k)+X1(k)*P(k)*TSCDCPnonresp(k))/N(k);

%%The estimates for the children meeting the cutoff for broader ASD classification based on the ADOS were similarly calculated, after accounting for the SCQ non-respondents using the method described above.

Q(k)=NADOSpos(k)*(NSCQpos(k)/NADOS(k))/NSCQ(k); % Rate within SCQ respondents after weighting for ados non respondents

Z(k)=(Q(k)*NSCQ(k)+ X2(k)*Q(k)*NSCQnonresp(k))/NPSCDC(k); % Rate after weighting for SCQ non respondents

Final_estimate_ados(k)= (Q(k)*NSCQ(k)+X2(k)*Q(k)*NSCQnonresp(k)+X1(k)*Z(k)*TSCDCPnonresp(k))/N(k);%Rate after weighting PSCDC non respondents

end

% Calculating mean and confidence interval after bootstrap

alpha=0.05;

U_Mean_final_scq=mean(UP_SCQ); % Unweighted SCQ prevalence

ci_u_scq=prctile(UP_SCQ,[100*alpha/2,100*(1-alpha/2)]);

U_Mean_final_ados=mean(UP_ADOS);% Unweighted ADOS prevalence

ci_u_ados=prctile(UP_ADOS,[100*alpha/2,100*(1-alpha/2)]);

Mean_final_scq=mean(Final_estimate_supra_SCQ); % Supra-threshold SCQ prevalence

ci_final_scq=prctile(Final_estimate_supra_SCQ,[100*alpha/2,100*(1-alpha/2)]);

Mean_final_ados=mean(Final_estimate_ados);% Broader autism ados prevalence

ci_final_ados=prctile(Final_estimate_ados,[100*alpha/2,100*(1-alpha/2)]);
